# Fast Hearing-Threshold Estimation Using Multiple Auditory Steady-State Responses with Narrow-Band Chirps and Adaptive Stimulus Patterns

**DOI:** 10.1100/2012/192178

**Published:** 2012-04-24

**Authors:** Roland Mühler, Katrin Mentzel, Jesko Verhey

**Affiliations:** Department of Experimental Audiology, Otto-von-Guericke University Magdeburg, Leipziger Street 44, 39120 Magdeburg, Germany

## Abstract

This paper describes the estimation of hearing thresholds in normal-hearing and hearing-impaired subjects on the basis of multiple-frequency auditory steady-state responses (ASSRs). The ASSR was measured using two new techniques: (i) adaptive stimulus patterns and (ii) narrow-band chirp stimuli. ASSR thresholds in 16 normal-hearing and 16 hearing-impaired adults were obtained simultaneously at both ears at 500, 1000, 2000, and 4000 Hz, using a multiple-frequency stimulus built up of four one-octave-wide narrow-band chirps with a repetition rate of 40 Hz. A statistical test in the frequency domain was used to detect the response. The recording of the steady-state responses was controlled in eight independent recording channels with an adaptive, semiautomatic algorithm. The average differences between the behavioural hearing thresholds and the ASSR threshold estimate were 10, 8, 13, and 15 dB for test frequencies of 500, 1000, 2000, and 4000 Hz, respectively. The average overall test duration of 18.6 minutes for the threshold estimations at the four frequencies and both ears demonstrates the benefit of an adaptive recording algorithm and the efficiency of optimised narrow-band chirp stimuli.

## 1. Introduction

Auditory steady-state responses (ASSRs) are commonly evoked by amplitude-modulated continuous tones. The often shown correlation between ASSR amplitude and stimulus presentation level can be used to objectively estimate hearing thresholds in infants as well as in adults and handicapped individuals [1, 2]. The presence or absence of a response can be determined by objective detection algorithms on the basis of statistical test in the frequency domain [3]. For clinical applications, ASSRs are usually evoked by stimuli modulated at rates near 40 Hz or 80 Hz. Although ASSRs for a 40-Hz modulation rate are up to four times larger than those for 80 Hz [4], the latter modulation rate is preferred for the estimation of hearing thresholds in infants. One reason for this practice is related to the effect of sedation, anaesthesia, and sleepiness on the 40 Hz responses [1]. Furthermore, ASSRs were used to investigate the effect of aging on temporal coding in the auditory system [5]. 

Several studies showed that ASSR for the 40 Hz and 80 Hz modulation rates accurately estimate the degree and configuration of the hearing loss in both adults and infants [6–10]. An interaction between the degree of hearing loss and the accuracy of ASSR threshold estimation was observed with smaller differences between behavioural and ASSR thresholds in subjects with a sensorineural hearing loss than in normal-hearing subjects [11, 12]. 

The attractiveness of ASSR for clinical applications mostly results from the possibility to be recorded simultaneously for multiple frequencies to one or both ears [13, 14], reducing the clinical testing time considerably. Common multiple-frequency ASSR systems present all stimulus components at the same stimulation level. However, since response amplitudes depend on the hearing loss at the tested frequency, it is unlikely that the responses to the different frequencies of the multiple-frequency stimulus reach significance at the same time [11, 15]. Thus the duration of each multiple-frequency ASSR recording will therefore be determined by the test frequency with the smallest amplitude or, in the worst case, by the time the algorithm needs to decide, where at this particular presentation level and frequency no response can be detected. To overcome the problem of unequal response amplitudes, John et al. [15] proposed an independent adjustment of intensity levels for each frequency component of the stimulus. Mühler et al. [16] showed results of an implementation of this method in a laboratory study. However, to the authors' knowledge, there is no report in the literature describing the use of a recording technique for multiple-frequency ASSR using adaptive stimulus patterns under clinical conditions. 

ASSR elicited by sinusoidally amplitude-modulated continuous tones show rather small amplitudes. To detect these small responses at stimulation intensities near the individual hearing threshold, long recording times are necessary [17]. Consequentially, several studies aimed to increase the ASSR amplitude by combining amplitude and frequency modulation [18] or modifying the shape of the envelope [19, 20]. However, none of these methods yielded a substantial increase of the response amplitude. Recently, Elberling et al. [21] proposed the application of chirp stimuli for the recording of steady-state responses. Chirp stimuli have been described by Lütkenhöner et al. [22] and Dau et al. [23] for the recording of auditory brainstem responses (ABRs), compensating for the traveling wave delay of the frequency components of a click stimulus at the basilar membrane. They argued that such a compensation results in a higher temporal synchronization of the neural structures contributing to the ABR, producing remarkably large response amplitudes. By constructing narrow-band chirps with an octave bandwidth, Elberling et al. [24] were able to adopt the chirp technique to the multiple-frequency concept of the ASSR.

The aim of the present study is twofold: On the one hand, this study investigates how accurate multiple-frequency ASSR with one-octave-band chirps can predict typical audiometric configurations of hearing loss, and on the other hand, the clinical value of multiple-frequency ASSR using adaptive stimulus patterns in normal-hearing adults and in adults with mild and moderate degrees of sensorineural hearing loss is evaluated.

## 2. Methods

### 2.1. Subjects

Two groups of adult subjects participated in this study: 16 subjects with normal hearing (NH) and 16 subjects with hearing impairment (HI). For the purposes of this study, NH was defined as thresholds of 20 dB HL or better at all audiometric frequencies between 500 and 4000 Hz, and HI was defined as thresholds of >30 dB HL at least one audiometric frequencies between 500 and 4000 Hz. The sample of individual audiograms covers flat configuration losses as well as gradual high- and low-frequency sloping losses. All hearing losses were of sensorineural origin. Behavioural thresholds were obtained with a clinical audiometer (Interacoustics AC40) in a sound-insulated booth. The age range for the NH subjects (10 female and 6 male) was 20–64 years with a mean of 36.8 years, and the age range for the HI subjects (6 female and 10 male) was 29–76 years with a mean of 50.9 years. Both ears were tested and included in the analysis. The protocol used in this study was in accordance with the Declaration of Helsinki. It was approved by the Ethics Review Board of the Otto-von-Guericke-University Magdeburg, and all subjects provided written informed consent.

### 2.2. Recording Parameters

All data were collected with a commercial ASSR software module (Interacoustics, version 1.02) running on an Interacoustics Eclipse EP25 platform. Subjects were placed on a comfortable couch in a sound-insulated and electrically shielded booth and were instructed to relax but not to sleep. Ag/AgCl electrodes were placed at the vertex (+) and both earlobes (−) with a ground electrode at the forehead. Impedances were kept below 5 kOhms. The EEG activity was amplified by 80 dB, bandpass filtered from 0.5 Hz to 5 kHz and digitized with a 16-bit resolution, and an artefact rejection level of ±40 *μ*V was applied. Acoustic stimuli were presented through ER-3A earphones.

### 2.3. Stimulation

ASSRs were recorded simultaneously from both ears with a multiple-stimulus paradigm as described by John et al. [14], known as MASTER (Multiple Auditory Steady-state Responses). In the current study, the stimuli presented to each of the two ears were generated by the superposition of four one-octave-wide chirps centred at 500, 1000, 2000, and 4000 Hz and with amplitude-frequency characteristics given in IEC 61260 [25]. Generation and properties of these narrow-band chirp stimuli have been described in detail by Elberling et al. [21, 24]. Calibration values of the four octave-band chirps for ER-3A earphones have been provided by the “Physikalisch-Technische Bundesanstalt” (Braunschweig, Germany) and are given by Elberling et al. [24].

To separate the responses elicited by these four stimuli in the EEG spectrum and to assign the responses to the correct test frequency and ear, each single stimulus was presented at a slightly different repetition rate, centred around 40 Hz (38, 41, 35, and 39 Hz for the right ear and 43, 45, 42, and 44 Hz for the left ear).

### 2.4. Adaptive Recording Algorithm

In contrast to other commercially available multiple-stimulus ASSR recording systems [26], the software running on the ASSR system used in the current study allows not only for a simultaneous but also for an independent threshold estimation at four test frequencies at the right and four test frequencies at the left ear. This is achieved by choosing the stimulus presentation level for each of the test frequencies independently and by running independent response detection algorithms in the eight recording channels.

After having been started, the algorithm seeks for a significant steady-state response in each of these recording channels. For this purpose, the EEG was transformed to the frequency domain by means of a fast Fourier transformation (FFT). The residual noise of each recording was determined online by averaging the noise value in the frequency bins surrounding the eight response bins.

A modified Rayleigh test, including both amplitude and phase information from the fundamental frequency and from higher harmonics, was used to detect the response [3]. If the critical test value has reached the level of significance, the algorithm stops the recording in this particular channel, whereas recording in the remaining channels continues. Within the software module used in the current study, the user can choose between two levels of significance (*P* = 0.05 and *P* = 0.01), representing a “fast” and an “accurate” recording mode. All data reported in this study were recorded using the “fast” mode.

When after a recording time of six minutes the response was not significant, the algorithm stopped the recording, suggesting a “no-response” decision. When the residual noise of this single recording was below 40 nV, the “no-response” decision was accepted by the operator; otherwise the recording time was prolonged until the noise level had reached 40 nV.

When a response was detected, the stimulus presentation level for this particular test frequency and ear was decreased manually by 10 dB; otherwise the stimulus presentation level was increased by 10 dB. By increasing or decreasing the stimulus presentation level of only one frequency component, the level pattern of the multiple-frequency stimulus approximates the frequency-specific audiogram for one ear step by step. To avoid masking effects, the maximum level difference between adjacent frequencies was limited to 20 dB.

The recording session was finished when at least one “response-present” and one “no-response” condition have been reached for all test frequencies at both ears. ASSR thresholds were defined as the lowest intensity where a response was present and a no-response was obtained at 10 dB lower.

### 2.5. Data Analysis

The differences between the behavioural thresholds and the ASSR thresholds were calculated for the four test frequencies and for both ears. These threshold differences were compared by a three-way repeated measures mixed ANOVA with the factors test frequency (500, 1000, 2000, and 4000 Hz), ear and hearing loss (NH and HI). Degrees of freedom were corrected using the Greenhouse-Geisser estimates of sphericity. Residual noise levels measured for the “no-response” condition were compared with an ANOVA in the same manner.

## 3. Results

**Figure 1:**
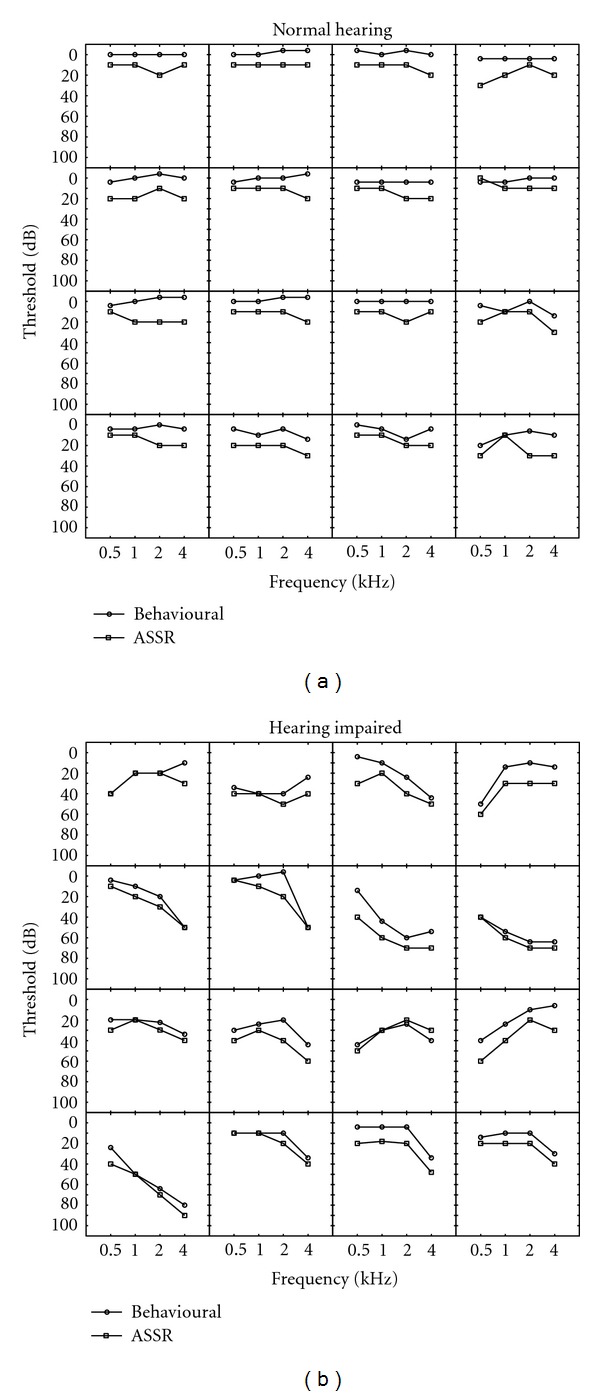
Behavioural and multiple ASSR thresholds at 500, 1000, 2000, and 4000 Hz plotted for the right ears of 16 normal-hearing subjects (a) and 16 hearing-impaired subjects (b).

**Figure 2:**
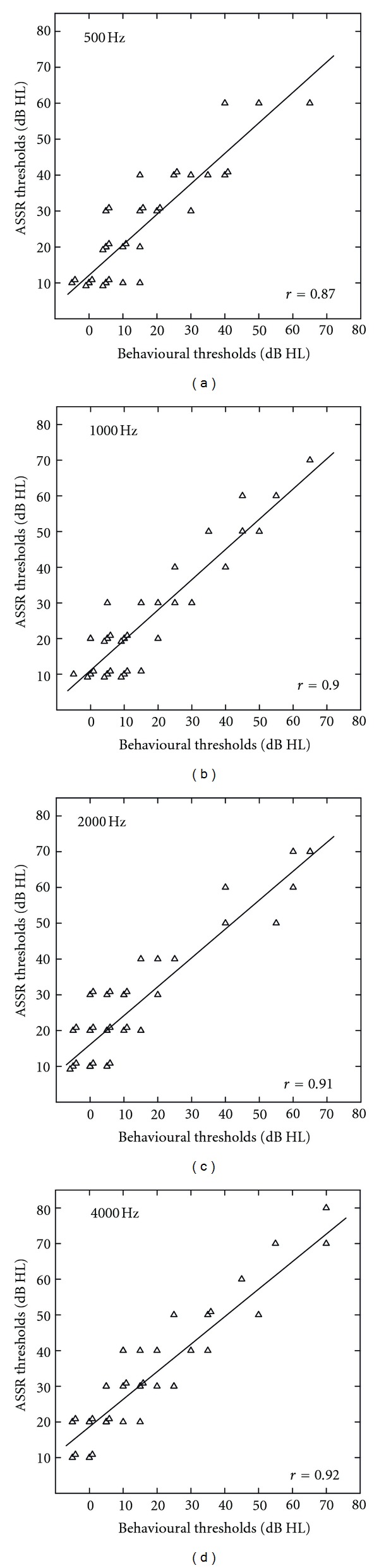
Linear regression analysis comparing ASSR thresholds with behavioural pure-tone thresholds at 500, 1000, 2000, and 4000 Hz with the correlation coefficient *r* in the lower right-hand corner of each plot. Overlapping data points are shifted by ±1 dB in both directions to improve the readability of the figure.

Individual behavioural and ASSR thresholds for the right ear of all 16 subjects from the NH and the HI group are plotted in Figure 1, respectively. Visual inspection of these audiograms shows that, in general, the ASSR thresholds follow the shape of the hearing loss.  Figure 2 shows scatterplots representing the linear regression analysis comparing behavioural and ASSR thresholds at 500, 1000, 2000, and 4000 Hz. The correlation coefficients ranged from 0.87 for 500 Hz to 0.92 for 4000 Hz (*P* < 0.001) indicating that the two threshold estimates were significantly correlated.

**Figure 3:**
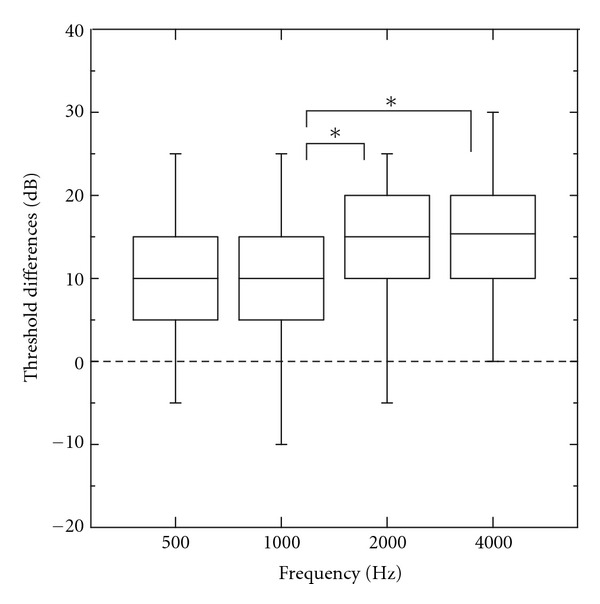
Differences between multiple ASSR thresholds and behavioural pure-tone thresholds for 32 subjects at 500, 1000, 2000, and 4000 Hz. Outer limits of each box represent the 25th and 75th percentiles, with the median shown as the line within the box. Whiskers indicate the 5th and 95th percentiles. (Bonferroni's post hoc tests: *P < 0.05).

The differences between the behavioural and ASSR thresholds are listed in Table 1 for both the NH and HI group.  Figure 3 summarizes these data collapsed for both groups and both ears. The ANOVA revealed a main effect for the test frequency (*F*(2.63,78.9) = 7.17, *P* < 0.001), and no effect for the hearing loss (*F*(1,30) = 4.1, *P* = 0.052) and for the ear tested (*F*(1,30) < 1). Post hoc comparisons (Bonferroni) revealed significant larger threshold differences for 2000 and 4000 Hz as compared to 1000 Hz (*P* < 0,01).

**Figure 4:**
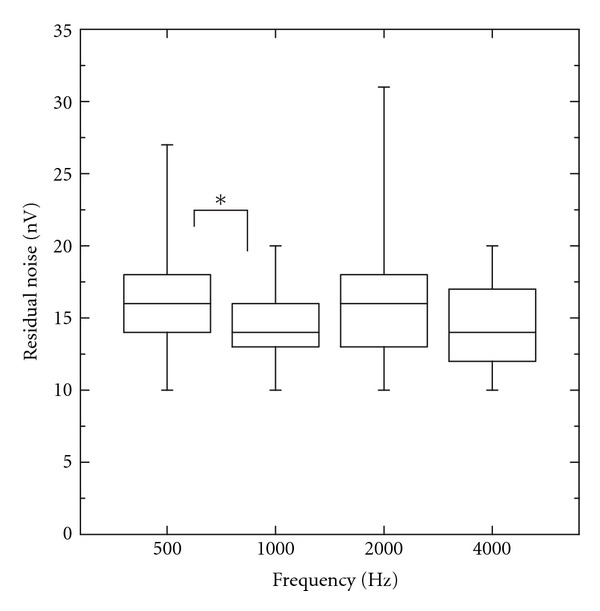
Residual noise of the test runs which were used for the “no-response” decision at four test frequencies for 32 subjects. Outer limits of each box represent the 25th and 75th percentiles, with the median shown as the line within the box. Whiskers indicate the 5th and 95th percentiles. (Bonferroni's post hoc tests: *P < 0.05).

Figure 4 provides an overview of the residual noise values for a recording time of 6 minutes which were used for the “no-response” decision. Mean noise values were found between 14 and 17 nV. The ANOVA revealed no main effects for the ear (*F*(1,30) < 1) and for the hearing loss (*F*(1,30) < 1) but a weak effect for the test frequency (*F*(2.412,72.35) = 3.12, *P* = 0.041). Post hoc comparisons (Bonferroni) revealed significant lower residual noise for 1000 Hz as compared to 500 Hz (*P* < 0.05).

**Figure 5:**
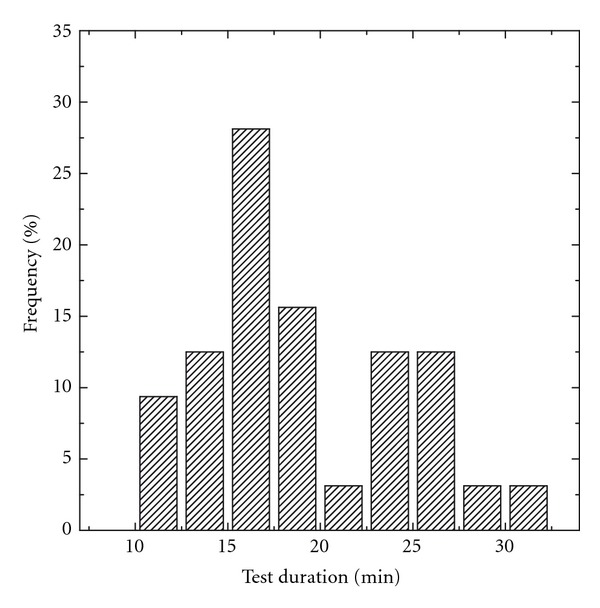
Distribution of total test duration for hearing-threshold estimations in 32 subjects (16 normal hearing, 16 hearing impaired) using multiple ASSR with narrow-band chirps and adaptive stimulus patterns.

The total recording time for the threshold estimation at four frequencies for both ears, obtained with the semi-automatic adaptive algorithm, was found between 10 and 31 minutes and was on average 18.6 minutes (standard deviation of 5.4 minutes). A distribution of the total recording times is provided in Figure 5. On average, thresholds for the NH subjects were obtained faster (16.1 ± 5.0 minutes) than in HI subjects (21.2 ± 6.6 minutes). This difference was significant as revealed by an independent *t*-test (*t*(30) = −3.05, *P* < 0.001).

## 4. Discussion

The aims of this study were (i) to demonstrate that narrow-band chirp stimuli with a one-octave bandwidth can be used to estimate hearing thresholds in adults using a multiple-frequency ASSR paradigm and (ii) to check the feasibility of a semiautomatic adaptive recording algorithm for ASSR.

The quality of a frequency-specific estimation of hearing thresholds with evoked potentials can be evaluated using two criteria: a “visual” and a “numerical.” The “visual” congruence between the individual ASSR thresholds and the corresponding behavioural thresholds of the NH and HI participants of the current study is very good from the clinician's point of view. Similar data from individual subjects have been reported by Herdman and Stapells [11] and Van Maanen and Stapells [27]. 

The one-octave-band chirps used in this study have a much broader spectrum than the amplitude-modulated stimuli used in the majority of the previous studies. Using these stimuli in a multiple-frequency (MASTER) paradigm, attention should be paid to a possible interaction between the stimulus components. For amplitude-modulated sinusoidal stimuli, these interactions have been investigated in detail by John et al. [14]. The aim of the present study was not specifically to evaluate masking effects between one-octave-band chirps which were used in a multifrequency paradigm. Nevertheless, the good agreement between the behavioural audiograms and the ASSR audiograms in the present study indicates that masking, at least to a clinical relevant extent, did not occur. One reason for this finding is presumably the constraints for the levels of adjacent frequencies: stimulus-level differences between neighbouring frequencies greater than 20 dB were not accepted by the algorithm.

The numerical differences between behavioural and ASSR thresholds of about 10 to 19 dB for NH subjects and 8 to 12 dB for HI subjects (Table 1) are in reasonable agreement with those reported in other multiple ASSR studies with 40 Hz stimulus modulation rate [2, 12, 27] and with 80 Hz stimulus modulation rate [7, 28, 29]. Van Maanen and Stapells [27] report mean threshold differences between 4 dB and 17 dB for 80 Hz ASSR and between 1 dB and 13 dB for 40 Hz ASSR, measured in subjects with several configurations of sensorineural hearing loss. D'haenens et al. [7] recorded 80 Hz ASSR at 500, 1000, 2000, and 4000 Hz in normal-hearing subjects and in subjects with mild and moderate sensorineural hearing loss. They found mean threshold differences between 10 dB and 19 dB in normal-hearing subjects and between 9 dB and 14 dB in subjects with mild and moderate sensorineural hearing loss, respectively.

Significant larger threshold differences were found in the present data for test frequencies of 2000 and 4000 Hz as compared to 1000 Hz. It is possible that calibration issues may account for this discrepancy. For clinical purposes it is essential that an objective procedure based on evoked potential reproduces the individual shape of the audiogram correctly. A more or less constant offset between behavioural and evoked potential thresholds can be corrected by empirically determined correction values.

One important factor affecting the accuracy of the threshold estimation with ASSR is the residual noise of the recording. The reliability of a “no-response” decision depends critically on the residual noise level [28], which, on the other hand, is not only determined by the recording time but also by the EEG amplitude, which in turn is influenced by the subject's state of arousal [30]. Therefore the critical noise level and the maximum recording time are crucial parameters of each automated or semiautomated ASSR recording algorithm. The maximum recording time of six minutes, which was preset in the system used in the current study, turned out to be adequate for clinical purposes, since all critical residual noise levels measured in our subjects were below 40 nV (Figure 4). This is considerably lower than the critical noise level of 60 nV proposed by Van Maanen and Stapells [27] for 40Hz ASSR. The mean residual noise levels from our recordings between 14 and 17 nV are even lower than the 20 nV used by Dimitrijevic et al. [8] and Herdman and Stapells [11] for the 80 Hz ASSR.

The short total recording times between 10 and 31 minutes with a mean at 18.6 minutes for the threshold estimation at four frequencies and both ears demonstrate the efficacy of the semiautomatic adaptive algorithm. When comparing these durations with those reported in the literature, it should be kept in mind that the present data were recorded in normal hearing and mildly to moderately hearing-impaired subjects. The significant longer recording times for the HI group show that even with adaptive stimulus patterns as used in the present study, sloping audiograms require more iteration steps. In a test-retest study with 29 normal hearing subjects, D'haenens et al. [17] used a nonadaptive descending threshold search protocol with a maximum recording time per intensity of 8 to 15 minutes, resulting in a total test duration of 1 hour and 20 minutes. Such recording times of 15 minutes per intensity step result in very low residual noise levels between 3.3 and 6.7 nV. For clinical purposes, such recording times are, however, not acceptable. Van Maanen and Stapells [27] reported a mean test duration of 20.4 minutes for a threshold estimation with multiple-frequency 40 Hz ASSR at four frequencies in one ear. Mean total recording times for threshold estimation with multiple 80 Hz ASSR reported by Herdman and Stapells [11] were between 44 and 49 minutes for hearing-impaired subjects with steep-sloping or flat-sloping audiograms, respectively. Using a similar recording system, D'haenens et al. [28] report total test durations between 43 and 46 minutes. Taking into account that the mean recording time of the present study represents simultaneously recorded threshold data from both ears, the benefit of an adaptive recording algorithm and the efficiency of optimised narrow-band chirp stimuli are evident.

The present study supports the findings of other groups, showing that multiple-frequency 40 Hz ASSRs accurately predict behavioural audiograms in adults with normal hearing and moderate sensorineural hearing loss. The use of optimised octave-band chirp stimuli and a semi-automatic adaptive recording algorithm reduces the total test duration considerably.

## Figures and Tables

**Table 1:** Differences between multiple ASSR thresholds and behavioural pure-tone thresholds for the normal-hearing (NH) and hearing-impaired (HI) test group (means and standard deviations, data from both ears collapsed).

	Frequency/Hz	Threshold difference/dB
NH	500	11,7 ± 7,9
	1000	9,7 ± 7,4
	2000	15,2 ± 7,5
	4000	18,9 ± 5,9

HI	500	10,6 ± 9,6
	1000	8,1 ± 8,6
	2000	12,0 ± 7,8
	4000	10,9 ± 9,8
